# Pulley traction strategy during esophageal endoscopic submucosal dissection: a new way to optimize submucosal exposure

**DOI:** 10.1055/a-2569-0931

**Published:** 2025-04-11

**Authors:** Timothée Wallenhorst, Mathieu Pioche, Thomas Grainville, Mael Pagenault, Louis Jean Masgnaux, Jérémie Jacques, Fabien Pinard

**Affiliations:** 1 36684 Department of Endoscopy and Gastroenterology, University Hospital Centre Rennes, Rennes, France; 2 Gastroenterology and Endoscopy Unit, Edouard Herriot Hospital, Hospices Civils de Lyon, Lyon, France; 3 Gastroenterology and Endoscopy Unit, Dupuytren University Hospital, Limoges, France; 4 Gastroenterology and Endoscopy Unit, Cornouaille Hospital, Quimper, France

Esophageal endoscopic submucosal dissection (ESD) is the gold standard for the treatment of superficial esophageal cancers. Japanese guidelines suggest the systematic use of traction, but there is no standardized strategy. Use of the “tunnel + clip” strategy by less experienced operators produced results of high proficiency 1 . Pulley methods have been described for early-stage gastric cancer 2 and recently for colorectal locations 3 , but no further studies have been made in the esophageal location.

We report the case of 67-year-old woman with Barrett’s esophagus. Magnifying endoscopy with acetic acid chromoendoscopy showed a target lesion of early-stage adenocarcinoma justifying an ESD. We decided to combine the “tunnel + clip” strategy with the pulley method.

As shown in Video 1 , after marking the lesion, we performed a hemicircumferential incision at the anal side and then at the oral side. The scope was withdrawn completely. A hemostatic clip was inserted into the operating channel and attached to a 250-cm-long line. The scope was reinserted parallel to the line, and the clip with the attached line was used to grasp the mucosa of the proximal tunnel entry. A line loop was passed over the original line and fixed with a clip on the gastric wall ( Fig. 1 ). Constant traction was applied by grasping the external part of the line with 11G Kocher forceps. Dissection was performed with optimized submucosal exposure. Pathology analysis revealed complete R0 resection of a Barrett’s esophagus segment 60 × 30 mm in size with a 40-mm intramucosal adenocarcinoma.

Pulley traction strategy during esophageal endoscopic submucosal dissection: a new way to optimize submucosal exposure.Video 1

**Fig. 1:**
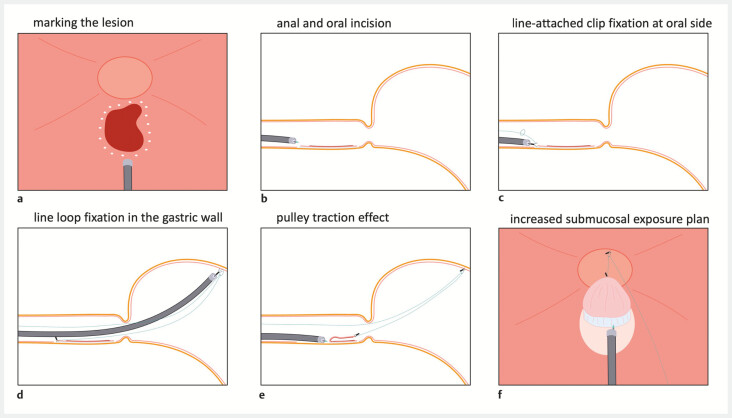
Schematic representation of esophageal endoscopic submucosal dissection using the “tunnel + clip” strategy combined with the pulley method. **a** Marking the lesion. **b** Anal and oral incision. **c** Line-attached clip fixation at the oral side. **d** Line loop fixation in the gastric wall. **e** Pulley traction effect. **f** Increased submucosal exposure.

The pulley traction strategy for esophageal ESD offers optimized submucosal exposure and could provide an additional traction tool to facilitate the procedure. Further studies are needed, especially with low-experience operators performing esophageal ESD.

Endoscopy_UCTN_Code_TTT_1AO_2AG_3AD

## Notes

Endoscopy E-Videos https://eref.thieme.de/e-videos
*E-Videos* is an open access online section of the journal *Endoscopy* , reporting on interesting cases and new techniques in gastroenterological endoscopy. All papers include a high-quality video and are published with a Creative Commons CC-BY license. Endoscopy E-Videos qualify for HINARI discounts and waivers and eligibility is automatically checked during the submission process. We grant 100% waivers to articles whose corresponding authors are based in Group A countries and 50% waivers to those who are based in Group B countries as classified by Research4Life (see: https://www.research4life.org/access/eligibility/ ). This section has its own submission website at https://mc.manuscriptcentral.com/e-videos .
